# *In Utero* and Childhood Polybrominated Diphenyl Ether (PBDE) Exposures and Neurodevelopment in the CHAMACOS Study

**DOI:** 10.1289/ehp.1205597

**Published:** 2012-11-15

**Authors:** Brenda Eskenazi, Jonathan Chevrier, Stephen A. Rauch, Katherine Kogut, Kim G. Harley, Caroline Johnson, Celina Trujillo, Andreas Sjödin, Asa Bradman

**Affiliations:** 1 Center for Environmental Research and Children’s Health, School of Public Health, University of California at Berkeley, Berkeley, California, USA; 2 Division of Laboratory Sciences, National Center for Environmental Health, Centers for Disease Control and Prevention, Atlanta, Georgia, USA

**Keywords:** ADHD, attention, biomarkers, children, cognitive development, flame retardants, human exposure, intelligence quotient, Mexican, motor, neurodevelopment, prenatal

## Abstract

background: California children’s exposures to polybrominated diphenyl ether flame retardants (PBDEs) are among the highest worldwide. PBDEs are known endocrine disruptors and neurotoxicants in animals.

Objective: Here we investigate the relation of *in utero* and child PBDE exposure to neurobehavioral development among participants in CHAMACOS (Center for the Health Assessment of Mothers and Children of Salinas), a California birth cohort.

Methods: We measured PBDEs in maternal prenatal and child serum samples and examined the association of PBDE concentrations with children’s attention, motor functioning, and cognition at 5 (*n* = 310) and 7 years of age (*n* = 323).

Results: Maternal prenatal PBDE concentrations were associated with impaired attention as measured by a continuous performance task at 5 years and maternal report at 5 and 7 years of age, with poorer fine motor coordination—particularly in the nondominant—at both age points, and with decrements in Verbal and Full-Scale IQ at 7 years. PBDE concentrations in children 7 years of age were significantly or marginally associated with concurrent teacher reports of attention problems and decrements in Processing Speed, Perceptual Reasoning, Verbal Comprehension, and Full-Scale IQ. These associations were not altered by adjustment for birth weight, gestational age, or maternal thyroid hormone levels.

Conclusions: Both prenatal and childhood PBDE exposures were associated with poorer attention, fine motor coordination, and cognition in the CHAMACOS cohort of school-age children. This study, the largest to date, contributes to growing evidence suggesting that PBDEs have adverse impacts on child neurobehavioral development.

Polybrominated diphenyl ether (PBDEs) flame retardant chemicals, used in the manufacture of furniture, infant products, and electronics, are ubiquitous in U.S. households (Sjödin et al. 2008). An unintended consequence of California’s Technical Bulletin 117 (TB 117)—a fire safety law promulgated in the 1970s which requires that furniture, baby, and other household products resist open flame (California Department of Consumer Affairs 2000; Zota et al. 2008)—is that PBDE concentrations in California children are now among the highest measured worldwide (Eskenazi et al. 2011). Until 2005, the predominant chemical flame retardant used to comply with TB 117 was pentaBDE (comprising congeners BDEs 47, 99, 100, and 153). Although pentaBDE was banned in California and phased out by the manufacturer in 2004, pentaBDEs continue to leach from older household items. Exposure is also perpetuated by decaBDEs, still used in many electronic products, which can break down into lower-brominated congeners (Noyes et al. 2011). Because PBDEs are semivolatile and not chemically bound to substrates, they migrate into house dust, placing young children, who crawl on the floor and exhibit frequent hand-to-mouth behaviors, at risk of higher exposures (Stapleton et al. 2008).

PBDEs are endocrine-disrupting compounds with half-lives in humans ranging from 2 to 12 years (Geyer et al. 2004). Recent research suggests that PBDE exposures are associated with altered thyroid hormone levels in pregnant women (Chevrier et al. 2010) and infants (Herbstman et al. 2008), and negatively associated with birth weight (Harley et al. 2011). Research also suggests possible neurotoxic effects of *in utero* and early childhood exposure to PBDEs (Chao et al. 2007; Gascon et al. 2011, 2012; Herbstman et al. 2010; Hoffman et al. 2012; Roze et al. 2009). Herbstman et al. (2010) reported significant decrements in motor and mental development at ages 1–6 years associated with *in utero* PBDE exposures in New York children (*n* = 100). In a study of 62 5- to 6-year-old Dutch children, Roze et al. (2009) reported that *in utero* exposure levels were negatively associated with fine motor coordination and sustained attention, although improved coordination and visual perception and fewer internalizing and externalizing behaviors. Recently, Gascon et al. (2011) reported that 4-year-old Spanish children with detectable blood concentrations of BDE-47 were significantly more likely to demonstrate attention symptoms [DSM-IV (*Diagnostic and Statistical Manual of Mental Disorders*, 4th ed.) (American Psychiatric Association 1994) scores > 80th percentile] than less-exposed peers, but not motor or cognitive deficits. Cord blood BDE-47 concentrations were not associated with any neurobehavioral parameters at 4 years of age. Hoffman et al. (2012) found a positive association between breast milk levels of BDEs 47, 99, and 100 and externalizing behaviors, specifically activity/impulsivity behaviors in 220 30-month-olds.

In this analysis, we examined the relationship of prenatal maternal and child PBDE concentrations with attention, cognition, and motor development in California children at 5 and 7 years of age.

## Methods

The Center for the Health Assessment of Mothers and Children of Salinas (CHAMACOS) is a longitudinal birth cohort study of predominantly Mexican-American families in California’s Salinas Valley. Detailed methods for CHAMACOS are published elsewhere (Eskenazi et al. 2004, 2006). Eligible pregnant women (≥ 18 years old, < 20 weeks gestation, Spanish- or English-speaking, qualifying for low-income health insurance, and planning to deliver at the public hospital) were recruited between October 1999 and October 2000 from community clinics. The cohort included 601 women, 526 of whom delivered live-born singletons.

Women were interviewed twice during pregnancy (at ~ 13 and 26 weeks gestation), after delivery, and when children were 6 months old, and 1, 2, 3.5, 5, and 7 years old. Mothers completed the Peabody Picture Vocabulary Test (PPVT) or Test de Vocabulario en Imágenes Peabody (TVIP) of verbal intelligence (Dunn and Dunn 1981) at the 6-month visit and the Center for Epidemiologic Studies Depression Scale (CES-D) (Radloff 1977) at the 1-year visit. Age-appropriate versions of the HOME (Home Observation for Measurement of the Environment) survey were completed at most postdelivery visits (Caldwell and Bradley 1984). Birth weight and gestational duration were abstracted from medical records.

Neurobehavioral assessments were performed by bilingual psychometricians, and children were assessed in their dominant language. A total of 310 children were assessed at 5 years (mean = 60.0 ± 2.6 months) and 323 at 7 years (85.2 ± 2.9 months). The present analysis excludes four children with autism, Down syndrome, cerebral palsy/hydrocephalus, or deafness and 63 children who lacked PBDE measurements.

Compared with children in the cohort who were not followed, children included in the present analyses were more likely to be female and born full term, with mothers who were older, breastfed longer, and were less likely to smoke or drink during pregnancy (data not shown). They did not differ according to other sociodemographic characteristics or by their maternal prenatal PBDE levels [median = 24.9 ng/g lipid; interquartile range (IQR) 14.0–42.1] for those followed versus 23.8 ng/g lipid (IQR = 14.9–41.3) for those not followed].

Mothers provided written informed consent at both visits, and children provided verbal assent at 7 years of age. Study activities were approved by the University of California at Berkeley (UC) Committee for the Protection of Human Subjects. A technical assistance agreement was established between the Division of Laboratory Sciences at the National Center for Environmental Health, Centers for Disease Control and Prevention (CDC), and UC Berkeley.

*Attention.* At the 5-year visit, mothers completed the Child Behavior Checklist (CBCL)/1.5–5 (CBCL) (Achenbach and Rescorla 2000). We analyzed two subscales as continuous raw scores: the Attention Problems scale and the DSM-IV–oriented Attention Deficit/Hyperactivity Disorder (ADHD) Problems scale. We also analyzed a “borderline clinical range” (≥ 93rd percentile in the standardization sample) indicator variable for each scale (Achenbach and Rescorla 2000). In addition, children were assessed on the Conners’ Kiddie Continuous Performance Test (K-CPT) (Conners and Staff 2001), a 7-min computerized vigilance task that assesses reaction time, accuracy, and impulse control. We determined continuous *T*-scores (standardized to a nonclinical population) for errors of commission, errors of omission, and hit reaction time (Conners and Staff 2001). We also examined the continuous ADHD Confidence Index score, which indicates the probability that children are correctly classified as having clinical ADHD, and a binary variable indicating a Confidence Index score ≥ 70th percentile.

At child’s age 7 years, mothers and teachers completed the Conners’ ADHD/DSM-IV Scales (CADS) (Conners 2001) and the Behavior Assessment System for Children, 2nd edition (BASC) (Reynolds and Kamphaus 2004). CADS data from four subscales (Conners ADHD index score, and DSM-IV–based Inattentive, Hyperactive/Impulsive, and Total ADHD scores) were analyzed both as continuous, standardized scores (*T*-scores; mean ± SD = 50 ± 10) and as a binary variable indicating scores in the “Moderately” or “Markedly Atypical” range (*T*-score ≥ 65) (Conners 2001). BASC data from Hyperactivity and Attention Problems subscales were analyzed as standardized *T*-scores and as a binary “at-risk” or “clinically significant” variable (*T*-score ≥ 60) (Reynolds and Kamphaus 2004).

*Motor function.* At ages 5 and 7 years, children’s gross motor skills were assessed using select subscales of the McCarthy Scales of Children’s Abilities (McCarthy 1972). Their fine motor dexterity was assessed with a pegboard test (Wide Range Assessment of Visual Motor Ability; WRAVMA) (Adams and Sheslow 1995) (age-standardized mean = 100 ± 15) and with a finger-tapping task [at 5 years: Behavioral Assessment and Research System (BARS) (Rohlman et al. 2003); and at 7 years: Reitan Neuropsychology Laboratory (Tucson, AZ)]. We standardized McCarthy gross motor and finger tap scores within our study population (*z*-scores, mean = 0 ± 1).

*Cognitive functioning.* At 5 years of age, children completed tests of receptive verbal intelligence in both English and Spanish using the PPVT and TVIP, respectively (Dunn and Dunn 1981). We analyzed children’s continuous standardized scores (mean = 100 ± 15) in their language of best performance. We assessed children’s performance intelligence (PIQ) with the Wechsler Preschool and Primary Scale of Intelligence, 3rd edition (WPPSI-III) (mean = 100 ± 15).

At age 7 years, children were assessed on four subdomains of the Wechsler Intelligence Scale for Children–Fourth Edition (WISC-IV) (Wechsler 2003): Verbal Comprehension, Perceptual Reasoning, Working Memory, and Processing Speed. A Full-Scale IQ was also calculated (mean = 100 ± 15 for the Full-Scale IQ and all components).

*Other questions.* Mothers were also asked “Has a doctor, nurse, psychologist or teacher ever told you that your child might have *1*) attention problems? or *2*) learning problems?” Teachers were asked “Do you have any specific concerns about this student (in terms of) *1*) emotional problems, *2*) behavioral problems, or *3*) learning problems?”

*PBDE exposure assessment.* Blood samples were collected by venipuncture from mothers during pregnancy (mean = 26.7 ± 2.6 weeks gestation, *n* = 219) or at delivery (*n* = 60), and from children at the 7-year visit (*n* = 272). PBDE serum levels in women with data at both time points were very strongly correlated (Pearson *r* ≥ 0.98, *p* < 0.001)]. Samples were immediately processed and stored at –80^o^C until shipment on dry ice to the CDC (Atlanta, GA). Samples were analyzed at CDC for 10 congeners (BDEs 17, 28, 47, 66, 85, 99, 100, 153, 154, and 183) using gas chromatography isotope dilution high-resolution mass spectrometry (Sjödin et al. 2004). PBDE concentrations are expressed on a serum lipid basis (nanograms per gram lipids). Total serum lipid concentrations were determined based on the measurement of triglycerides and total cholesterol using standard enzymatic methods (Roche Chemicals, Indianapolis, IN) (Phillips et al. 1989). The limits of detection (LODs) for BDE-47 ranged from 0.3 to 2.6 ng/g lipids for maternal samples, and 0.4 to 0.8 ng/g lipids for child samples. For all other congeners, LODs ranged between 0.2 and 0.7 ng/g lipids for maternal and 0.3 and 5.6 ng/g lipids for child samples, respectively. Quality control samples (blanks and spikes) were included in each run.

We used the sum of BDEs 47, 99, 100, and 153 congeners as our primary exposure measure. Values < LOD were assigned the machine-read value if a signal was detected. If not, all concentration levels < LOD were imputed at random based on a log-normal probability distribution using maximum likelihood estimation (Lubin et al. 2004).

We assessed maternal exposure to organophosphate (OP) insecticides as measured by dialkyl phosphate (DAP) metabolites in maternal urine (at 13 and 26 weeks gestation), using an isotope dilution gas chromatography-tandem mass spectrometry method (Bradman et al. 2005; Bravo et al. 2002); lead in maternal prenatal and cord blood samples, using graphite furnace atomic absorption spectrophotometry; polychlorinated biphenyls (PCBs) in maternal serum using high-resolution gas chromatography/high-resolution mass spectrometry with isotope dilution quantification (Barr et al. 2003); and maternal thyroid stimulating hormone (TSH; using immunochemiluminometric assay) and free thyroxine (T_4_; using direct equilibrium dialysis followed by radioimmunoassay) (Bayer ADVIA Centaur system; Siemens Healthcare Diagnostics, Deerfield, IL) at 26 weeks gestation (Chevrier et al. 2010; Nelson and Tomei 1988).

*Data analysis.* PBDE levels were expressed on the log_10_ scale. To determine the shape of the dose–response function, we ran generalized additive models using cubic splines. If nonlinearity was detected (*p* < 0.10), additional models were run with categorized PBDE concentrations (quartiles). We re-ran all final models with PBDE concentrations expressed on a serum basis (picograms per gram serum) with total serum lipids as a covariate. We also ran models with the sum of all 10 PBDE congeners; individually for each of the four primary congeners (47, 99, 100, and 153); and excluding outliers (defined as being ≥ 3.5 SD away from the mean for log_10_ PBDEs or the outcome).

Variables were identified as potential confounders based on their relationship to neurodevelopment. We examined the following [see Supplemental Material, Table S3 for categories (http://dx.doi.org/10.1289/ehp.1205597)]: maternal age, education, years in the United States, marital status, work outside the home, use of alcohol and tobacco during pregnancy, depression (CES-D), parity, and PPVT or TVIP score; housing density, household poverty, pregnancy exposure to environmental tobacco smoke, number of children in the home, father’s presence in the home, and HOME score at 6 months and 7 years; preschool and out-of-home child care attendance; psychometrician, location, and language of assessment; and child sex, birth weight, preterm delivery status, and handedness (motor outcomes only). Missing values (< 10%) for covariates were imputed by randomly selecting a value from the dataset.

We built separate models for attention, cognition, and motor outcomes, and used the same model for all outcomes within a category. In addition to child’s sex and months of age (continuous), final models included all covariates that changed the coefficient for the main exposure and any outcome within the group by > 10%. The covariates maintained in the models are listed in the footnote of the respective tables.

For sensitivity analyses, we adjusted for birth weight, gestational age at birth, maternal thyroid hormone (TSH and free T_4_), DAPs, lead, and PCBs in separate models (Chevrier et al. 2010; Harley et al. 2011). We evaluated effect modification by child sex. In addition, we included maternal and child PBDE levels in the same models, although doing so reduced the sample size (*n* = 214).

Main effects were considered statistically significant with *p* < 0.05 based on two-tailed tests, and interactions were considered significant if *p* < 0.10. All analyses were conducted with STATA version 10.1 (StataCorp, College Station, TX).

## Results

For both mothers and children, BDE congeners 47, 99, 100, and 153 had detection frequencies > 97% and dominated the total measure of concentration, with BDE-47 in the highest concentration [for maternal and child measures, see Supplemental Material, Tables S1 and S2, respectively (http://dx.doi.org/10.1289/ehp.1205597)]. Children’s PBDE levels were more than three times higher than the mothers’ for the sum of four congeners, and detection frequencies for most other congeners were also substantially higher in children (Bradman et al. 2012; Castorina et al. 2011; Eskenazi et al. 2011). The correlation between maternal and child ΣPBDE levels was 0.27 (*p* < 0.001); the correlation for individual congeners ranged from 0.21 for BDE-99 to 0.30 for BDE-153. Supplemental Material, Table S3, presents the distribution of demographic characteristics for children in the study sample and the geometric means (GM) of maternal and child ΣPBDE concentrations by covariates. Supplemental Material, Table S4, summarizes neurobehavioral scores for the study population.

Correlations between reports by teachers and parents concerning attention at 7 years of age, and between measures of attention, cognition, and motor skills, were moderate. For example, correlations between maternal and teacher report on the CADS ranged from *r* = 0.2–0.3 (*p* < 0.01). Similar measures of attention on the BASC and CADS within a reporter (mother/teacher) were more strongly correlated—for example, *r* = 0.5 to 0.8, *p* < 0.001 for maternal report and *r* = 0.7 to 0.8, *p* < 0.001 for teacher report. Maternal and teacher CADS scores were negatively correlated with WISC Full-Scale IQ scores (*r* = –0.2 to –0.3, *p* < 0.001). Motor skills outcomes tended to be positively correlated with IQ scores (*r* = 0.1 to 0.4, several *p* < 0.001) and negatively correlated with attention outcomes (*r* = –0.05 to –0.2, several *p* < 0.01) (data not shown).

**Figure 1:**
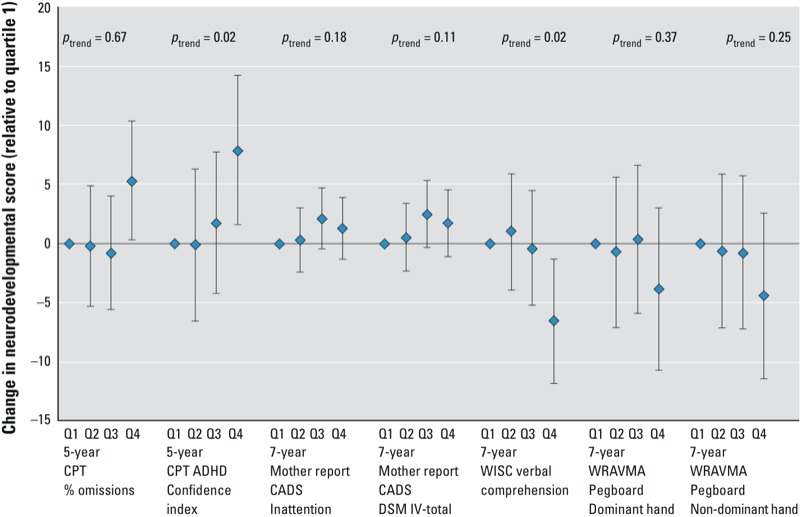
The point estimate and 95% CI for each quartile (Q) of maternal ∑PBDE concentration for outcomes that showed overall associations and evidence of nonlinearity (at *p *< 0.1). The quartile ranges for maternal PBDEs were ≤ 14.4, 14.5–24.78, 24.8–41.97, and ≥ 42 ng/g lipid. Tests for trend come from models using PBDE quartile (1–4) as a continuous variable.

*Attention.* At child age 5 years, maternal prenatal ΣPBDE concentrations (for the 4 main congeners) were marginally associated (*p* < 0.10) with maternally reported CBCL scores above the 93rd percentile for attention problems [adjusted odds ratio (aOR) for a 10-fold increase in ΣPBDE = 4.6; 95%CI: 0.9, 24.5] [see Supplemental Material, Table S5 (http://dx.doi.org/10.1289/ehp.1205597)], and strongly associated with both errors of omission scores and ADHD Confidence Index scores on the K-CPT (Table 1). Quartile categorization suggested that both errors of omission and the ADHD Confidence Index were primarily elevated in children with mothers in the highest quartile of ΣPBDE exposure (> 42 ng/g) (Figure 1).

**Table 1:** Adjusted linear models for attention-related outcome scores in CHAMACOS children at 5 and 7 years of age, per 10-fold increase in maternal prenatal and child ∑PBDE concentration (ng/g, lipid-adjusted).

Outcome	Maternal ∑PBDEa,c		Child ∑PBDEb,c	
	n	β (95% CI)	n	β (95% CI)
Assessment of 5-year-olds
CBCL (raw score)
Attention problems	249	0.1 (–0.4, 0.6)
ADHD	249	0.4 (–0.5, 1.2)
K-CPT (T-score)
Errors of omission	246	5.8 (1.5, 10.1)**,#
Errors of commission	246	–0.5 (–3.7, 2.7)
ADHD Confidence Index	233	7.0 (1.6, 12.4)**,#
Assessment of 7-year-olds
Conner’s rating scale (CADS)–maternal report (T-score)
ADHD index	266	2.9 (0.7, 5.2)**	270	1.0 (–1.9, 3.9)
DSM-IV total scale	266	2.6 (0.2, 5.0)**,#	270	1.4 (–1.5, 4.4)
Inattentive subscale	266	2.2 (0.0, 4.5)**,#	270	0.7 (–2.1, 3.5)#
Hyperactive/Impulsive subscale	266	1.6 (–0.8, 4.1)	270	1.9 (–1.1, 5.0)
BASC-2–maternal report (T-score)
Hyperactivity scale	257	1.0 (–1.5, 3.6)	269	0.5 (–2.6, 3.5)
Attention Problems scale	257	0.5 (–1.2, 2.1)	269	–0.1 (–2.1, 1.9)
Conner’s rating scale (CADS)–teacher report (T-score)
ADHD index	213	2.4 (–1.4, 6.1)	219	4.6 (–0.4, 9.6)*
DSM-IV total scale	212	1.8 (–1.4, 5.0)	217	4.0 (–0.3, 8.3)*
Inattentive subscale	216	1.2 (–1.6, 3.9)	221	3.7 (0.1, 7.4)**
Hyperactive/Impulsive subscale	216	1.7 (–1.7, 5.0)	221	3.5 (–1.1, 8.0)
BASC-2–teacher report (T-score)
Hyperactivity scale	217	1.8 (–1.3, 4.9)	222	4.8 (0.5, 9.0)**
Attention Problems scale	257	0.7 (–1.3, 2.7)	222	2.9 (0.4, 5.5)**
aMaternal PBDE models control for child’s age at assessment, sex, maternal education, number of children in the home, and psychometrician (5-year assessments only). bChild PBDE models control for child’s age at assessment, sex, and parity. cSum of four PBDE congeners: BDEs 47, 99, 100, and 153. *p < 0.1. **p ≤ 0.05. #Digression from linearity at p < 0.10.					

At child age 7 years, maternal ΣPBDE exposure was associated with maternally reported ADHD Index scores on the CADS (β = 2.9; 95% CI: 0.7, 5.2), DSM-IV Total scores (β = 2.6; 95% CI: 0.2, 5.0), and DSM-IV Inattention scale scores (β = 2.2; 95% CI: 0.0, 4.5) (Table 1). Although there was evidence of nonlinearity for the DSM measures, quartile categorization showed no clear trends (Figure 1). Maternal exposure was also related to somewhat higher odds of a mother having been told that her child had attention problems (aOR = 2.3; 95% CI: 0.9, 5.8), and to teacher reports of child behavior problems (aOR = 2.5; 95% CI: 1.1, 6.0) [see Supplemental Material, Table S5 (http://dx.doi.org/10.1289/ehp.1205597)]. However, there were no associations between maternal ΣPBDE and teacher ratings on the CADS or BASC, or maternal ratings on the BASC, for continuous or dichotomous outcomes.

By contrast, child PBDE concentrations were associated with reports of attention problems from teachers, but not from mothers. Specifically, child ΣPBDEs were associated with more adverse teacher reports on CADS ADHD Index, CADS DSM-IV Total, CADS DSM-IV Inattentive, BASC Hyperactivity, and BASC Attention Problems scales [Table 1; see also Supplemental Material, Table S5 (http://dx.doi.org/10.1289/ehp.1205597)]. Associations were particularly pronounced for some of the dichotomous outcomes: Every 10-fold increase in child ΣPBDE level was associated with 4.5 and 5.5 times higher odds of the child being rated by the teacher as being in the “moderately or markedly atypical” range on CADS DSM-IV Hyperactive/Impulsive subscale (95% CI: 1.2, 16.6) and DSM-IV Total subscale (95% CI: 1.5, 20.3), respectively (see Supplemental Material, Table S5).

*Motor function.* We observed little evidence of association between either maternal or child ΣPBDE serum concentrations and gross motor performance on McCarthy scales (Table 2). However, maternal ΣPBDEs were related to poorer performance on the WRAVMA pegboard at both 5 and 7 years, particularly for the nondominant hand. For the 5-year-olds, this relationship was observed primarily for the nondominant hand among boys (boys: β = –12.1; 95% CI: –19.4, –4.7; girls: β = 0.8; 95% CI: –6.8, 8.5; *p*_interaction_ = 0.09), whereas at age 7, it was seen mainly in the dominant hand in girls (boys: β = –2.7; 95% CI: –10.8, 5.4; girls β = –8.1; 95% CI: –16.3, 0.1; *p*_interaction_ = 0.08). Associations between maternal ΣPBDEs and pegboard performance at 7 years showed evidence of nonlinearity, with nonsignificantly poorer performance in children of mothers in the highest quartile of exposure (Figure 1). At 5 but not 7 years of age, maternal ΣPBDEs were also inversely associated with dominant-hand finger taps (Table 2).

**Table 2:** Adjusted linear models for motor function in CHAMACOS children at 5 and 7 years of age, per 10-fold increase in maternal prenatal and child ∑PBDE concentration (ng/g, lipid-adjusted).

Outcome	Maternal ∑PBDEa,b		Child ∑PBDEb,c	
	n	β (95% CI)	n	β (95% CI)
Assessment of 5-year-olds
WRAVMA pegboard (standard score)
Dominant hand	254	–4.3 (–9.6, 1.0)
Nondominant hand	252	–5.6 (–10.8, –0.4)**,##
Finger tap (BARS z-score)
Dominant hand	234	–0.4 (–0.7, 0.0)**
Nondominant hand	234	–0.2 (–0.5, 0.1)
McCarthy (z-score)
Gross motor leg	241	0.0 (–0.3, 0.4)#
Bean bag catch	249	–0.1 (–0.4, 0.2)#
Assessment of 7-year-olds
WRAVMA pegboard (standard score)
Dominant hand	258	–5.4 (–11.1, 0.3)*,#,##	269	–5.4 (–12.0, 1.2)
Nondominant hand	258	–6.5 (–12.3, –0.7)**,#	268	–6.1 (–12.7, 0.4)*
Finger tap (BARS z-score)
Dominant hand	258	–0.1 (–0.4, 0.2)#	269	–0.2 (–0.6, 0.2)
Nondominant hand	258	–0.1 (–0.4, 0.2)#	268	–0.1 (–0.5, 0.2)
McCarthy (z-score)
Gross motor leg	255	–0.1 (–0.4, 0.1)#	266	–0.1 (–0.4, 0.2)#
Bean bag catch	258	0.0 (–0.3, 0.4)	268	0.0 (–0.4, 0.3)
aMaternal PBDE models control for child’s age, sex, home score at 6-month visit, father living with family, handedness, location of testing, whether the child attended preschool, maternal years in United States before giving birth, and psychometrician (5-year assessment only). bSum of four PBDE congeners: BDEs 47, 99, 100, and 153. cChild PBDE models control for child’s age, sex, home score at 7-year visit, and location of testing. *p < 0.1. **p ≤ 0.05. #Digression from linearity at p < 0.10. ##Interaction with child sex at p < 0.10.					

Child ΣPBDEs were marginally related to nondominant hand pegboard performance at age 7 years, but not with other motor outcomes.

*Cognitive functioning.* We observed no associations between maternal ΣPBDE concentrations and child PPVT/TVIP or WPPSI Performance IQ scores at age 5 years (Table 3). However, at age 7 years, maternal ΣPBDEs were associated with significant decrements in WISC Verbal Comprehension IQ, contributing to a somewhat lowered Full-Scale IQ. Quartile analysis indicated that the association was primarily driven by a Verbal Comprehension IQ decrement in the highest quartile (β = –6.0; 95% CI: –11.3, –0.7; see Figure 1).

**Table 3:** Adjusted linear models for measures of cognition at 5 and 7 years of age (standard score), per 10-fold increase in maternal prenatal and child ∑PBDE concentration (ng/g, lipid-adjusted).

Outcome	Maternal ∑PBDEa,b		Child ∑PBDEb,c	
	n	β (95% CI)	n	β (95% CI)
Assessment of 5-year-olds
PPVT	252	0.4 (–5.1, 5.9)
Performance IQ	256	0.9 (–3.5, 5.3)
Assessment of 7-year-olds
Full-Scale IQ	231	–4.7 (–9.4, 0.1)*	248	–5.6 (–10.8, –0.3)**
Verbal Comprehension IQ	258	–5.5 (–10.0, –1.0)**,#	269	–4.3 (–9.4, 0.8)*
Perceptual Reasoning IQ	258	–2.4 (–7.6, 2.9)	269	–5.2 (–11.1, 0.7)*
Working Memory IQ	231	–2.4 (–7.2, 2.3)#	249	–2.3 (–7.4, 2.8)
Processing Speed IQ	232	–2.3 (–6.8, 2.3)	249	–6.6 (–11.4, –1.8)**
aMaternal PBDE models control for child’s age, sex, home score at 6-month visit, language of assessment, and maternal years living in United States before giving birth. bSum of four PBDE congeners: BDEs 47, 99, 100, and 153. cChild PBDE models control for child’s age, sex, home score at 7-year visit, maternal PPVT, language of examination, maternal years living in the United States before giving birth, parity, and prenatal exposure to environmental tobacco smoke. *p < 0.1. **p ≤ 0.05. #Digression from linearity at p < 0.10.					

Children’s ΣPBDE concentrations were also related to Full-Scale IQ at age 7 years (β = –5.6; 95% CI: –10.8, –0.3), particularly with the Perceptual Reasoning IQ, Processing Speed IQ, and Verbal Comprehension IQ subscales (Table 3).

*Sensitivity analyses.* The above relationships were not confounded by maternal lead, PCB, or OP pesticide exposures, or substantially altered when controlled (in separate models) for birth weight, gestational age, or prenatal thyroid hormones. Overall, associations with individual PBDE congeners or the sum of all 10 congeners [see Supplemental Maternal, Table S6 (http://dx.doi.org/10.1289/ehp.1205597)] were generally consistent with results for the sum of the four major congeners. Depending on the outcome, there were between 0 and 4 outliers with respect to either ΣPBDE concentrations or outcomes; excluding them did not substantively affect the results (data not shown). Except where noted, we did not find evidence of effect modification by child sex.

When both maternal and child ΣPBDE levels were entered into the same model (*n* = 214), associations were attenuated (data not shown) but child ΣPBDE levels were still associated with a borderline increase in teacher-reported scores for inattention on the BASC (β = 2.8; 95% CI: –0.2, 5.7) and maternal ΣPBDE levels were still associated with maternally-reported CADS DSM-IV Total scale scores (β = 2.6; 95% CI: –0.3, 5.5), decreased Verbal Comprehension IQ (β = –5.2; 95% CI: –10.4, 0.1) and Full-Scale IQ (β = –5.2; 95% CI: –10.6, 0.1), and lower nondominant hand pegboard scores (β = –6.5; 95% CI: –13.4, 0.3).

## Discussion

In the present study, we report associations between mothers’ prenatal serum concentrations of PBDEs and evidence of deficits in attention, fine motor coordination, and cognitive functioning (particularly verbal comprehension) in their children at ages 5 and/or 7 years. Despite only weak correlations between PBDE concentrations in maternal prenatal and child age 7 years blood, we found associations between cognition, motor function and attention with both maternal and child PBDE exposures. The observed results appeared to be independent of associations previously reported in this cohort between maternal PBDEs and maternal thyroid hormone (Chevrier et al. 2010) or child birth weight (Harley et al. 2011) and between maternal organophosphate pesticide exposure and child neurobehavioral development (Bouchard et al. 2011; Eskenazi et al. 2007; Marks et al. 2010). In addition, associations were not confounded by maternal lead or PCB levels, which were at low background levels.

This is the largest study to date on the potential neurodevelopmental impacts of PBDE exposures, and largely supports findings from three smaller studies, including those with substantially lower PBDE serum levels (Gascon et al. 2011; Herbstman et al. 2010; Hoffman et al. 2012; Roze et al. 2009). Our results are also similar to those reported between prenatal exposure to PCBs, which are chemically similar to PBDEs, and poorer attention and cognition or mental development in children (Grandjean et al. 2001; Jacobson and Jacobson 2003; Koopman-Esseboom et al. 1996; Rogan and Gladen 1991; Sagiv et al. 2012).

A notable finding of our study is that, in addition to *in utero* exposures, childhood PBDE concentrations were also associated with neurodevelopmental deficits. Although we hypothesized *a priori* that prenatal exposure would be more influential than postnatal exposure, the 7-year-olds’ average PBDE concentrations were much higher than those in their mothers during pregnancy; we attribute this difference in part to the lifetime residence of the children in California compared with mothers, many of whom were recent immigrants to California when their levels were measured (Eskenazi et al. 2011).

In animal studies, PBDE exposure has been associated with increased death of cerebellar granule cells, alterations in neuronal arachidonic acid release, and disruption of calcium homeostasis (Birnbaum and Staskal 2004). Other potential mechanisms include perturbations of the cholinergic neurotransmitter system, interference with cellular signaling (Viberg et al. 2002a, 2002b, 2003), and, because of PBDEs’ structural similarity to T_4_, effects on maternal thyroid hormone necessary for normal infant brain development (Darnerud et al. 2007; Richardson et al. 2008; Zhou et al. 2002). However, maternal thyroid hormone did not appear to explain the associations observed in our study population, as adding it to models did not measurably change the results.

Important strengths of the current study include its longitudinal design and use of comprehensive neurobehavioral assessments, which incorporate input from multiple informants. Limitations of this study are that we did not observe consistency in associations with PBDEs across informants for measures of attention (although their responses were moderately correlated), and we constructed numerous statistical models (although performance across domains was also moderately correlated), which increased the possibility of a chance finding. We also did not measure some higher-brominated compounds (e.g., BDE-209), which are present in decaBDE. Another study, however, indicates that BDE-209 represents a very small fraction of total serum PBDE concentrations in a different population of California children (Rose et al. 2010).

## Conclusions

This study’s finding of significant associations of both maternal prenatal and childhood PBDE exposures with poorer attention, fine motor coordination, and cognition in early school-age children contributes to the growing evidence of adverse associations between PBDE exposure and children’s neurobehavioral development. Although these results are of particular concern for California children, they are also relevant to other locations, many of which contain products manufactured to meet California’s standards. With the phaseout of pentaBDE, other flame retardants have been used to achieve compliance with TB 117. Additional research is needed to determine the potential child health consequences of these new chemical flame retardants.

## Supplemental Material

(156 KB) PDF
