# Supplementary material for: In Utero and Childhood Polybrominated Diphenyl Ether (PBDE) Exposures and Neurodevelopment in the CHAMACOS Study
Source: Environ Health Perspect. 2012 Nov 15;121(2):257–62. doi: 10.1289/ehp.1205597 (PMC3569691; doi:10.1289/ehp.1205597)
Supplement: (156 KB) PDF [file ehp.1205597.s001.pdf]

Supplemental Material: *In Utero* and Childhood Polybrominated Diphenyl Ether (PBDE) Exposures and Neurodevelopment in the CHAMACOS Study

Brenda Eskenazi[1], Jonathan Chevrier[1], Stephen A. Rauch[1], Katherine Kogut[1], Kim G. Harley[1], Caroline Johnson[1], Celina Trujillo[1], Andreas Sjodin[2], Asa Bradman[1]

[1]Center for Environmental Research and Children's Health (CERCH), School of Public Health, University of California at Berkeley, Berkeley, CA
[2] Division of Laboratory Sciences, National Center for Environmental Health, Centers for Disease Control and Prevention, Atlanta, GA

Contents:

Supplemental Table S1: Detection and concentrations of PBDE congeners in maternal serum 2

Supplemental Table S2: Detection and concentration of PBDE congeners in child serum collected at age 7 years of age. 3

Supplemental Table S3: Distributions of sociodemographic characteristics for the CHAMACOS children by maternal and child ∑PBDE serum concentrations..4

Supplemental Table S4: Means and standard deviations for attention, neurodevelopment, and motor outcomes in the CHAMACOS Cohort at 5- and 7-year visits 7

Supplemental Table S5: Adjusted logistic models for dichotomous attention-related outcomes in CHAMACOS children age 5 and 7 years 9

Supplemental Table S6: Results for models using the sum of 4 PBDE congeners (primary analysis), sum of 10 congeners, and individual congeners BDE-47, -99, -100, and -153 11

Supplemental Material, Table S1: Detection and concentrations of PBDE congeners in maternal serum

PBDEs	N[a]	LOD[b] range (ng/g)	Detection freq[c] (%)	GM (95% CI)[d] (ng/g)	Min (ng/g)	10th % (ng/g)	25th % (ng/g)	Median (ng/g)	75th % (ng/g)	90th % (ng/g)	Max (ng/g)
∑4 PBDE[e]	279	0.2-2.6	100.0[f]	26.3 (23.4, 29.6)	2.6	8.0	14.0	24.9	42.1	84.8	1293.7
∑10 PBDE[g]	279	0.2-2.6	100.0[f]	28.7 (25.7, 32.1)	4.2	9.2	14.9	27.1	45.1	90.0	1379.4
BDE-17	278	0.2–0.7	1.4	0.05 (0.04, 0.05)	<LOD	<LOD	<LOD	<LOD	<LOD	<LOD	2.78
BDE-28	278	0.2–0.7	51.1	0.38 (0.32, 0.45)	<LOD	<LOD	<LOD	0.4	1.1	2.2	24.1
BDE-47	279	0.3–2.6	99.3	15.8 (14.0, 17.8)	<LOD	4.6	8.1	15.7	26.6	51.8	761.0
BDE-66	278	0.2–0.7	14.0	0.15 (0.13, 0.17)	<LOD	<LOD	<LOD	<LOD	<LOD	0.4	10.1
BDE-85	279	0.2–0.7	43.0	0.37 (0.33, 0.42)	<LOD	<LOD	<LOD	<LOD	0.6	1.1	27.4
BDE-99	279	0.2–0.7	98.6	4.5 (4.0, 5.1)	<LOD	1.4	2.3	4.2	7.2	13.9	297.6
BDE-100	279	0.2–0.7	97.5	2.8 (2.5, 3.2)	<LOD	0.8	1.5	2.6	4.7	9.6	138.3
BDE-153	279	0.2–0.7	97.5	2.4 (2.1, 2.7)	<LOD	0.8	1.4	2.1	3.9	7.8	96.9
BDE-154	279	0.2–0.7	40.1	0.38 (0.34, 0.43)	<LOD	<LOD	<LOD	<LOD	0.6	1.2	20.6
BDE-183	279	0.2–0.7	26.3	0.31 (0.28, 0.33)	<LOD	<LOD	<LOD	<LOD	0.4	0.6	10.8

All measures are lipid-adjusted (ng/g lipids)
[a]Includes children participating in 5- or 7-year neurodevelopmental assessment, minus exclusions for serious conditions or multiple births.
[b]Limit of Detection.
[c]Detection frequency.
[d]Geometric mean and 95% Confidence interval.
[e]Sum of four PBDE congeners: BDE-47, -99, -100, and -153.
[f]Percentage of samples with at least one congener above the limit of detection.
[g]Sum of all 10 PBDE congeners: BDE-17, -28, -47, -66, -85, -99, -100, -153, -154, and -183.

Supplemental Material, Table S2: Detection and concentration of PBDE congeners in child serum collected at age 7 years of age.

PBDEs	N[a]	LOD[b] range (ng/g)	Detection freq[c] (%)	GM (95% CI)[d] (ng/g)	Min (ng/g)	10th % (ng/g)	25th % (ng/g)	Median (ng/g)	75th % (ng/g)	90th % (ng/g)	Max (ng/g)
∑4 PBDE[e]	270	0.3–5.6	100.0[f]	84.4 (76.8, 92.7)	5.8	31.4	49.9	84.6	129.7	228.1	1308.5
∑10 PBDE[g]	270	0.3–5.6	100.0[f]	90.9 (82.8, 99.7)	6.9	34.8	54.4	91.8	141.6	241.1	1385.5
BDE-17	270	0.3–5.6	35.2	0.55 (0.50, 0.60)	<LOD	<LOD	<LOD	<LOD	0.9	1.6	11.8
BDE-28	270	0.3–5.6	87.4	2.0 (1.8, 2.2)	<LOD	<LOD	1.3	2.1	3.5	5.6	23.2
BDE-47	270	0.4–0.8	100.0	47.3 (42.8, 52.3)	1.9	17.4	27.8	46.9	77.3	135.4	768.2
BDE-66	270	0.3–5.6	37.0	0.56 (0.51, 0.61)	<LOD	<LOD	<LOD	<LOD	0.9	1.5	6.8
BDE-85	270	0.3–5.6	64.1	0.94 (0.86, 1.04)	<LOD	<LOD	<LOD	0.9	1.6	2.6	14.6
BDE-99	270	0.3–5.6	99.6	11.1 (10.0, 12.4)	<LOD	3.7	6.2	10.6	19.2	36.1	218.2
BDE-100	270	0.3–5.6	100.0	10.7 (9,8, 11.8)	0.8	3.9	5.9	10.9	16.8	32.6	144.0
BDE-153	270	0.3–5.6	100.0	12.2 (11.1, 13.3)	0.9	4.9	6.8	11.5	19.9	32.6	263.1
BDE-154	270	0.3–5.6	77.8	1.2 (1.1, 1.4)	<LOD	<LOD	0.7	1.1	1.9	3.5	19.0
BDE-183	270	0.3–5.6	20.0	0.46 (0.43, 0.50)	<LOD	<LOD	<LOD	<LOD	<LOD	0.9	17.0

All measures are lipid-adjusted (ng/g lipids)

[a]Includes children participating in 5- or 7-year neurodevelopmental assessment, minus exclusions for serious conditions or multiple births.

[b]Limit of Detection.

[c]Detection frequency.

[d]Geometric mean and 95% Confidence interval.

[e]Sum of four PBDE congeners: BDE-47, -99, -100, and -153.

[f]Percentage of samples with at least one congener above the limit of detection.

[g]Sum of all 10 PBDE congeners: BDE-17, -28, -47, -66, -85, -99, -100, -153, -154, and -183.

Supplemental Material, Table S3: Distributions of sociodemographic characteristics for the CHAMACOS children by maternal and child ∑PBDE serum concentrations

Covariate	N (%)[a]	Maternal ∑PBDE[b] GM (95% CI)	Child ∑PBDE[b] GM (95% CI)
Total study population	336	26.3 (23.4, 29.6)	84.4 (76.8, 92.7)
Maternal age (at delivery)			
18-24	141 (42.0)	26.2 (21.8, 31.4)	80.1 (69.3, 92.5)
25-29	111 (33.0)	23.9 (20.1, 28.3)	93.2 (79.5, 109.2)
30-34	54 (16.1)	27.2 (18.4, 40.2)	85.7 (66.5, 110.4)
35-45	30 (8.9)	38.0 (24.7, 58.7)	72.4 (50.5, 103.8)
Marital status			
Not married	62 (18.5)	33.9 (25.0, 46.0)**	91.1 (73.8, 112.3)
Married/living as married	274 (81.5)	24.8 (21.9, 28.1)	83.0 (74.7, 92.2)
Maternal education			
≤ 6th grade	150 (44.6)	22.7 (19.4, 26.5)*	73.1 (64.6, 82.7)**
7th – 12th grade	117 (34.8)	28.3 (22.5, 35.4)	90.0 (76.1, 106.4)
High School grad	69 (20.5)	32.0 (24.9, 41.1)	102.2 (81.1, 128.8)
Maternal years in US			
≤ 1	73 (21.7)	14.1 (11.4, 17.5)**	77.2 (63.4, 94.1)**
2-5	88 (26.2)	28.9 (22.5, 37.2)	74.5 (62.3, 89.1)
6-10	85 (25.3)	28.9 (24.2, 34.6)	83.3 (69.4, 99.9)
11+	54 (16.1)	32.0 (24.8, 41.2)	87.0 (66.4, 113.9)
Entire life	36 (10.7)	50.0 (35.9, 69.6)	129.5 (98.3, 170.4)
Poverty category (pregnancy)			
<100% of FPL	211 (62.8)	26.0 (22.6, 30.0)	81.9 (73.7, 91.0)
>100-200% of FPL	125 (37.2)	26.9 (21.9, 32.9)	88.6 (74.1, 106.1)
Alcohol consumption during pregnancy			
Yes	73 (22.2)	30.2 (24.6, 37.0)	91.6 (78.5, 106.9)
No	256 (77.8)	25.7 (23.1, 28.6)	81.9 (73.0, 92.0)
Smoked during pregnancy			
Yes	14 (4.2)	35.4 (11.5, 108.9)	92.0 (62.8, 134.8)
No	322 (95.8)	26.1 (23.2, 29.3)	84.0 (76.2, 92.6)
ETS-exposed in household (pregnancy)			
Yes	30 (9.2)	31.2 (19.1, 51.1)	91.6 (70.8, 118.6)
No	295 (90.1)	25.7 (22.8, 29.0)	83.8 (75.6, 92.3)

Supplemental Material, Table S3 (continued): Distributions of sociodemographic characteristics for the CHAMACOS children by maternal and child ∑PBDE serum concentrations

Covariate	N (%)[a]	Maternal ∑PBDE[b] GM (95% CI)	Child ∑PBDE[b] GM (95% CI)
Parity			
0	110 (32.7)	24.0 (19.2, 30.0)	68.9 (58.9, 80.6)**
1+	226 (67.3)	27.6 (24.2, 31.6)	93.1 (82.9, 104.4)
Child sex			
Male	158 (47.0)	24.5 (20.6, 29.1)	88.0 (76.4, 101.4)
Female	178 (53.0)	27.9 (23.8, 32.7)	81.3 (71.7, 92.3)
Birthweight			
< 2,500g	12 (3.6)	36.4 (18.5, 71.7)	125.3 (67.7, 232.3)
2,500 and over	324 (96.4)	26.0 (23.1, 29.3)	83.4 (75.8, 91.7)
Preterm birth			
< 37 weeks	21 (6.3)	33.7 (21.4, 53.3)	81.0 (57.4, 114.2)
≥ 37 weeks	315 (93.8)	25.9 (22.9, 29.2)	84.7 (76.7, 93.4)
Breastfeeding[c]			
≤ 3 months	120 (35.7)	31.1 (25.1, 38.6)*	79.2 (67.7, 92.6)
3.1–11.9 months	114 (33.9)	25.8 (21.7, 30.6)	79.5 (68.3, 92.7)
≥ 12 months	102 (30.4)	22.4 (17.9, 28.0)	95.6 (79.9, 114.4)
Preschool attendance			
Yes	222 (69.6)	26.7 (23.1, 30.7)	85.2 (76.2, 95.3)
No	97 (30.4)	25.2 (20.0, 31.8)	82.4 (68.2, 99.6)
HOME Score (at 6 months)			
<31	139 (42.0)	20.3 (17.0, 24.2)**	82.9 (72.5, 94.8)
31.1–33.3	82 (24.8)	30.1 (23.5, 38.6)	79.3 (64.8, 97.1)
≥33.4	110 (33.2)	31.5 (25.9, 38.2)	89.8 (75.3, 107.0)
HOME Score (at 7 years)			
<17	98 (31.1)	24.5 (20.3, 29.5)	72.9 (63.0, 84.3)**
17-19	131 (41.6)	28.0 (23.2, 33.8)	80.8 (70.0, 93.2)
>19	86 (27.3)	25.0 (20.1, 31.2)	108.3 (88.0, 98.5)
Father lives with family (at 7 years)			
All the time	232 (73.7)	23.6 (20.8, 26.8)**	82.1 (73.4, 91.9)
Most of the time	13 (4.1)	37.0 (22.2, 61.6)	97.3 (61.6, 153.5)
Some of the time	18 (5.7)	25.7 (12.7, 51.8)	90.9 (65.3, 126.5)
Not at all	52 (16.5)	35.3 (26.1, 47.9)	89.6 (70.8, 113.3)

Supplemental Material, Table S3 (continued): Distributions of sociodemographic characteristics for the CHAMACOS children by maternal and child ∑PBDE serum concentrations

Covariate	N (%)[a]	Maternal ∑PBDE[b] GM (95% CI)	Child ∑PBDE[b] GM (95% CI)
Mother works outside of home (at 7 years)			
Yes	172 (53.3)	25.5 (21.7, 29.8)	82.6 (72.5, 94.1)
No	151 (46.8)	26.9 (22.7, 32.0)	86.5 (75.4, 99.2)
Number of Children in Home (at 7 years)[c]			
1	33 (10.2)	22.4 (13.9, 36.1)	56.1 (40.2, 78.5)**
2	92 (28.5)	22.7 (18.2, 28.2)	77.2 (65.8, 90.5)
3	114 (35.3)	28.4 (23.6, 34.1)	81.2 (69.6, 94.9)
4 or more	84 (26.0)	29.4 (23.7, 36.4)	109.8 (90.8, 132.8)
Housing density (persons per room at 7 years)			
≤0.5	1 (0.3)	3.6 (N/A)	68.5 (N/A)
0.51 – 1.00	107 (33.0)	28.2 (23.3, 34.2)	95.8 (72.2, 102.0)
1.01 – 1.50	136 (42.0)	25.1 (21.1, 30.0)	79.9 (68.9, 92.6)
≥1.51	80 (24.7)	27.6 (21.4, 35.5)	85.8 (71.0, 103.8)
Primary language of assessment (at 5 years)			
Spanish	285 (92.3)	24.4 (21.6, 27.5)**	79.1 (71.8, 87.1)**
English	25 (7.7)	52.6 (33.1, 83.6)	171.0 (118.7, 246.1)
Primary language of assessment (at 7 years)			
Spanish	210 (66.7)	22.3 (19.4, 25.6)**	77.8 (70.0, 86.6)**
English	105 (33.3)	35.1 (29.2, 42.4)	100.1 (83.5, 120.1)

[a]Children who completed the 5- or 7-year neuropsychological assessment and had maternal *or* child PBDEs.
[b]Sum of 4 PBDE congeners (BDE-47, -99, -100, and -153)
[c]Analyzed in multivariate models as a continuous variable.
*p<0.1; **p≤0.05; p-values are for one-way ANOVAs across categories.

Supplemental Material, Table S4: Means and standard deviations for attention, neurodevelopment, and motor outcomes in the CHAMACOS cohort at 5- and 7-year visits

Outcome	N	Mean ± SD
**Assessment of 5-year olds**		
Attention Outcomes		
K-CPT, T-scores		
Errors of Omission	300	54.2 ± 14.4
Errors of Commission	300	54.3 ± 10.1
ADHD Confidence Index	285	45.9 ± 17.7
CBCL, Raw Scores		
Attention Problems	301	2.4 ± 1.6
ADHD	301	4.6 ± 2.9
Cognitive Outcomes, Standard Scores		
PPVT	305	94.6 ± 17.2
Performance IQ	310	95.6 ± 14.4
Motor Outcomes		
WRAVMA Pegboard, Standardized Scores		
Dominant Hand	307	111.6 ± 17.3
Non-dominant Hand	305	111.5 ± 16.8
Finger Tap, Raw Score		
Dominant Hand	285	47.8 ± 7.7
Non-dominant Hand	285	41.8 ± 6.4
McCarthy Gross Motor Tests, Raw Score		
Gross Motor Leg	291	11.0 ± 2.1
Bean Bag Catch	303	4.2 ± 2.4
**Assessment of 7-year olds**		
Attention Outcomes		
Conner's Rating Scale (CADS) – Maternal Report, T-score		
ADHD Index	323	49.9 ± 7.9
DSM-IV ADHD Scale	323	50.3 ± 8.3
Inattentive Subscale	323	48.9 ± 7.7
Hyperactive/Impulsive Subscale	323	51.9 ± 8.6

Supplemental Material, Table S4 (continued): Means and standard deviations for attention, neurodevelopment, and motor outcomes in the CHAMACOS cohort at 5- and 7-year visits

Outcome	N	Mean ± SD
Conner's Rating Scale (CADS) – Teacher Report, T-score		
ADHD Index	260	53.5 ± 11.6
DSM-IV ADHD Scale	259	52.0 ± 10.0
Inattentive Subscale	263	48.5 ± 8.8
Hyperactive/Impulsive Subscale	263	52.2 ± 10.5
BASC-2 – Maternal Report, T-score		
Hyperactivity	313	45.6 ± 8.5
Attention Problems	313	56.4 ± 5.4
BASC-2 – Teacher Report, T-score		
Hyperactivity	264	49.6 ± 10.2
Attention Problems	264	52.9 ± 6.2
Cognitive Outcomes		
Full-Scale IQ, Scaled Scores	285	104.0 ± 14.3
Verbal Comprehension IQ	315	106.4 ± 16.9
Perceptual Reasoning IQ	315	102.8 ± 16.2
Working Memory IQ	286	93.6 ± 13.6
Processing Speed IQ	286	108.4 ± 13.0
Motor Outcomes		
WRAVMA Pegboard, Scaled Scores		
Dominant Hand	315	121.0 ± 18.0
Non-dominant Hand	314	123.7 ± 18.0
Finger Tap, Raw Scores[a]		
Dominant Hand	315	32.4 ± 6.3
Non-dominant Hand	314	28.7 ± 5.6
McCarthy Gross Motor Tests, Raw Score[a]		
Gross Motor Leg	311	12.4 ± 1.0
Bean Bag Catch	314	4.9 ± 2.0

[a]For statistical analyses, these were converted to Z-scores for the CHAMACOS population.

Supplemental Material, Table S5: Adjusted logistic models for dichotomous attention-related outcomes in CHAMACOS children ages 5 and 7 years: odds ratios per 10-fold increase in maternal prenatal and child ∑PBDE concentration (ng/g, lipid-adjusted)

	Maternal ∑PBDE[a,c]		Child ∑PBDE[b,c]	
	Cases/N	OR (95% CI)	Cases/N	OR (95% CI)
**Assessment of 5-year olds**				
Child Behavior Checklist[d]				
Attention problems	8/249	4.6 (0.9, 24.5) *		
ADHD	16/249	1.7 (0.5, 5.9)		
K-CPT				
ADHD Confidence Index ≥70%	21/233	3.4 (1.2, 9.5) **		
**Assessment of 7-year olds**				
Conner's Rating Scale (CADS) – Maternal Report[e]				
ADHD Index	15/266	3.0 (1.0, 8.9) **	16/270	1.9 (0.4, 8.4)
DSM-IV ADHD scale	13/266	1.8 (0.5, 6.1)	13/270	1.9 (0.3, 10.7)
Inattentive subscale	11/266	1.6 (0.4, 6.0)	9/270	0.8 (0.1, 6.2)
Hyperactive/Impulsive subscale	15/266	1.8 (0.6, 5.8)	16/270	2.2 (0.5, 10.6)
BASC-2 – Maternal Report[f]				
Hyperactivity scale	11/257	1.9 (0.5, 8.3)	17/269	1.7 (0.4, 7.3)
Attention problems scale	46/257	1.7 (0.7, 3.7)	46/269	0.8 (0.3, 2.0)
Conner's Rating Scale (CADS) – Teacher Report[e]				
ADHD Index	35/213	1.7 (0.7, 4.1)	41/219	2.3 (0.8, 6.8)
DSM-IV Total scale	23/212	1.1 (0.4, 3.3)	29/217	5.5 (1.5, 20.3) **
Inattentive subscale	12/216	1.5 (0.4, 6.0)	14/221	3.7 (0.6, 21.9)
Hyperactive/Impulsive subscale	23/216	1.5 (0.6, 4.3)	29/221	4.5 (1.2, 16.6) **
BASC-2 – Teacher Report[f]				
Hyperactivity scale	28/217	1.1 (0.4, 3.0)	34/222	3.4 (1.0, 11.7) *
Attention problems scale	38/217	1.5 (0.6, 3.4)	46/222	3.6 (1.2, 10.4) **
Parent report – ever told child has:				
Attention problems	25/266	2.3 (0.9, 5.8) *	31/270	1.5 (0.5, 4.6)
Learning problems	34/266	1.1 (0.5, 2.7)	37/270	0.8 (0.3, 2.1)
Teacher report – does child have:				
Emotional problem	36/203	0.7 (0.3, 1.8)	36/208	0.9 (0.3, 2.9)
Behavioral problem	45/199	2.5 (1.1, 6.0) **	60/206	1.5 (0.6, 4.0)
Learning problem	64/198	1.7 (0.8, 3.6)	64/204	0.9 (0.4, 2.4)

Supplemental Material, Table S5 (continued): Adjusted logistic models for dichotomous attention-related outcomes in CHAMACOS children ages 5 and 7 years: odds ratios per 10-fold increase in maternal prenatal and child ∑PBDE concentration (ng/g, lipid-adjusted)

Abbreviations: ADHD = attention deficit hyperactivity disorder; CI = confidence interval; PBDE = polybrominated diphenyl ethers, K-CPT = Kiddie Continuous Performance Test
[a]Maternal PBDE models control for age, sex, maternal education, number of children in the home, and psychometrician (5-year assessments only).
[b]Child PBDE models control for age, sex, and parity.
[c]Sum of four PBDE congeners: BDE-47, -99, -100, and -153.
[d]Score in “borderline” or “clinical” range.
[e]Score in “moderately atypical” or “markedly atypical” range.
[f]Score in “at risk” or “clinically significant” range.
$^{*}p<0.1$; $^{**}p\leq 0.05$

Supplemental Material, Table S6: Results for models using the sum of 4 PBDE congeners (primary analysis), sum of 10 congeners, and individual congeners BDE-47, -99, -100, and -153: βs and 95% CIs for a 10-fold increase in the congener(s) indicated.

	Σ4 Congeners	Σ10 Congeners	BDE-47	BDE-99	BDE-100	BDE-153
**ATTENTION**						
**Maternal PBDEs**						
K-CPT (5 years)						
Errors of Omission	5.8 (1.5, 10.1)**	5.9 (1.5, 10.4)**	5.1 (1.0, 9.3)**	6.6 (2.6, 10.6)**	4.2 (0.0, 8.4)*	4.5 (0.0, 8.9)*
ADHD Conf. Index	7.0 (1.6, 12.4)**	7.2 (1.7, 12.8)**	6.2 (1.1, 11.4)**	7.2 (2.1, 12.2)**	5.7 (0.4, 10.9)**	5.8 (0.2, 11.3)**
Mother-reported CADS (7 years)						
ADHD Index	2.9 (0.7, 5.2)**	3.1 (0.7, 5.4)**	2.6 (0.4, 4.8)**	3.0 (0.9, 5.0)**	2.9 (0.7, 5.1)**	3.1 (0.6, 5.5)**
DSM-IV ADHD	2.6 (0.2, 5)**	2.7 (0.3, 5.2)**	2.2 (0.0, 4.5)*	2.7 (0.6, 4.9)**	2.6 (0.2, 4.9)**	2.5 (-0.1, 5.0)*
DSM-IV Inattentive	2.2 (0.0, 4.5)**	2.4 (0.1, 4.7)**	1.9 (-0.2, 4.0)*	2.5 (0.4, 4.5)**	2.1 (-0.1, 4.3)*	2.1 (-0.3, 4.5)*
DSM-IV Hyperactive	1.6 (-0.8, 4.1)	1.7 (-0.8, 4.2)	1.3 (-1.0, 3.7)	1.9 (-0.4, 4.1)*	1.7 (-0.7, 4.2)	1.7 (-1.0, 4.3)
**Child PBDEs**						
Teacher-Reported CADS (7 years)						
ADHD Index	4.6 (-0.4, 9.6)*	4.7 (-0.4, 9.7)*	3.7 (-1.0, 8.4)	3.3 (-1.0, 7.7)	4.5 (-0.3, 9.3)*	4.5 (-0.4, 9.4)*
DSM-IV ADHD	4.0 (-0.3, 8.3)*	4.1 (-0.3, 8.4)*	3.1 (-0.9, 7.2)	2.7 (-1.1, 6.4)	3.8 (-0.3, 7.9)*	4.1 (-0.2, 8.3)*
DSM-IV Inattentive	3.7 (0.1, 7.4)**	3.8 (0.0, 7.5)**	2.9 (-0.5, 6.4)*	2.5 (-0.7, 5.7)	3.4 (-0.1, 6.9)*	3.9 (0.3, 7.5)**
Teacher-Reported BASC (7 years)						
Hyperactivity	4.8 (0.5, 9.0)**	4.9 (0.6, 9.2)**	4.0 (0, 8.1)**	3.6 (-0.1, 7.3)*	4.6 (0.5, 8.7)**	3.9 (-0.3, 8.1)*
Attention Problems	2.9 (0.4, 5.5)**	3.0 (0.3, 5.6)**	2.6 (0.2, 5.1)**	2.4 (0.1, 4.6)**	2.6 (0.1, 5.1)**	2.0 (-0.6, 4.6)
**COGNITION**						
**Maternal PBDEs**						
Full-scale IQ (7 years)	-4.7 (-9.4, 0.1)*	-4.8 (-9.7, 0.0)*	-3.8 (-8.3, 0.7)	-4.5 (-8.6, -0.3)**	-3.9 (-8.7, 0.8)	-5.5 (-10.6, -0.3)**
Verbal Comp	-5.5 (-10, -1.0)**	-5.7 (-10.3, -1.1)**	-4.6 (-8.9, -0.3)**	-4.9 (-8.9, -0.9)**	-5.9 (-10.5, -1.4)**	-6.2 (-11.2, -1.1)**
**Child PBDEs**						
Full-scale IQ (7 years)	-5.6 (-10.8, -0.3)**	-5.6 (-10.9, -0.3)**	-4.2 (-9.2, 0.7)*	-4.5 (-9.0, 0.0)*	-5.3 (-10.4, -0.3)**	-6.8 (-12.0, -1.6)**
Verbal Comp	-4.3 (-9.4, 0.8)*	-4.4 (-9.5, 0.8)*	-3.5 (-8.2, 1.3)	-3.8 (-8.1, 0.6)*	-4.7 (-9.6, 0.2)*	-5.1 (-10.2, 0.1)*
Perceptual Resoning	-5.2 (-11.1, 0.7)*	-5.2 (-11.2, 0.8)*	-4.4 (-9.9, 1.1)	-4.8 (-9.8, 0.3)*	-5 (-10.7, 0.7)*	-4.2 (-10.2, 1.8)
Working Memory	-2.3 (-7.4, 2.8)	-2.3 (-7.5, 2.9)	-0.7 (-5.5, 4.1)	-1.3 (-5.7, 3.1)	-2.0 (-7.0, 2.9)	-5.7 (-10.8, -0.6)**
Processing Speed	-6.6 (-11.4, -1.8)**	-6.7 (-11.5, -1.8)**	-5.5 (-10, -1.0)**	-5.0 (-9.2, -0.9)**	-5.7 (-10.3, -1.0)**	-7.2 (-12.0, -2.4)**

Supplemental Material, Table S6 (continued): Results for models using the sum of 4 PBDE congeners (primary analysis), sum of 10 congeners, and individual congeners BDE-47, -99, -100, and -153: βs and 95% CIs for a 10-fold increase in the congener(s) indicated.

	Σ4 Congeners	Σ10 Congeners	BDE-47	BDE-99	BDE-100	BDE-153
**MOTOR**						
**Maternal PBDEs**						
WRAVMA Pegboard (5 years)						
Dominant Hand	-4.3 (-9.6, 1.0)	-4.3 (-9.8, 1.2)	-3.5 (-8.6, 1.6)	-4.6 (-9.3, 0.1)*	-3.9 (-9.1, 1.4)	-4.4 (-10.2, 1.4)
Non-dominant Hand	-5.6 (-10.8, -0.4)**	-5.6 (-11, -0.3)**	-5.0 (-10.0, 0.0)*	-5.4 (-10.1, -0.8)**	-5.5 (-10.6, -0.4)**	-5.6 (-11.2, 0.1)*
Finger-Tap z-Score (5 years)						
Dominant Hand	-0.4 (-0.7, 0.0)**	-0.4 (-0.7, 0.0)**	-0.3 (-0.6, 0.0)*	-0.4 (-0.7, -0.1)**	-0.3 (-0.7, 0.0)*	-0.3 (-0.6, 0.1)
WRAVMA Pegboard (7 years)						
Dominant Hand	-5.4 (-11.1, 0.3)*	-6.0 (-11.9, -0.2)**	-3.6 (-9.0, 1.9)	-6.7 (-11.7, -1.6)**	-5.4 (-11.1, 0.3)*	-6.1 (-12.5, 0.2)*
Non-dominant Hand	-6.5 (-12.3, -0.7)**	-7.1 (-13, -1.1)**	-4.8 (-10.4, 0.7)*	-7.6 (-12.7, -2.5)**	-6.1 (-11.9, -0.3)**	-6.2 (-12.7, 0.2)*
**Child PBDEs (7 years)**						
Dominant Hand	-5.4 (-12.0, 1.2)	-5.5 (-12.2, 1.2)	-4.2 (-10.4, 2.0)	-5.3 (-11.0, 0.4)*	-5.9 (-12.2, 0.5)*	-3.9 (-10.5, 2.7)
Non-dominant Hand	-6.1 (-12.7, 0.4)*	-6.3 (-12.9, 0.4)*	-5.3 (-11.4, 0.9)*	-5.8 (-11.4, -0.1)**	-6.3 (-12.6, 0.0)**	-3.8 (-10.4, 2.7)

Abbreviations: ADHD = attention deficit hyperactivity disorder; CI = confidence interval; PBDE = polybrominated diphenyl ethers; K-CPT = Kiddie Continuous Performance Test; CADS = Conners' ADHD/DSM-IV Scales; BASC= Behavior Assessment Scale for Children; WRAVMA= Range Assessment of Visual Motor Ability Maternal and child PBDE models control for same covariates as primary model (sum of 4 congeners).
*p<0.1; **p<0.05
